# Supplementary material for: Venomix: a simple bioinformatic pipeline for identifying and characterizing toxin gene candidates from transcriptomic data
Source: PeerJ. 2018 Jul 31;6:e5361. doi: 10.7717/peerj.5361 (PMC6074769; doi:10.7717/peerj.5361)
Supplement: Supplemental Information 6 [file peerj-06-5361-s006.gz › FinalOutput_1E-20/Disintegrin-like_leberagin-C_8/finaltree.pdf]

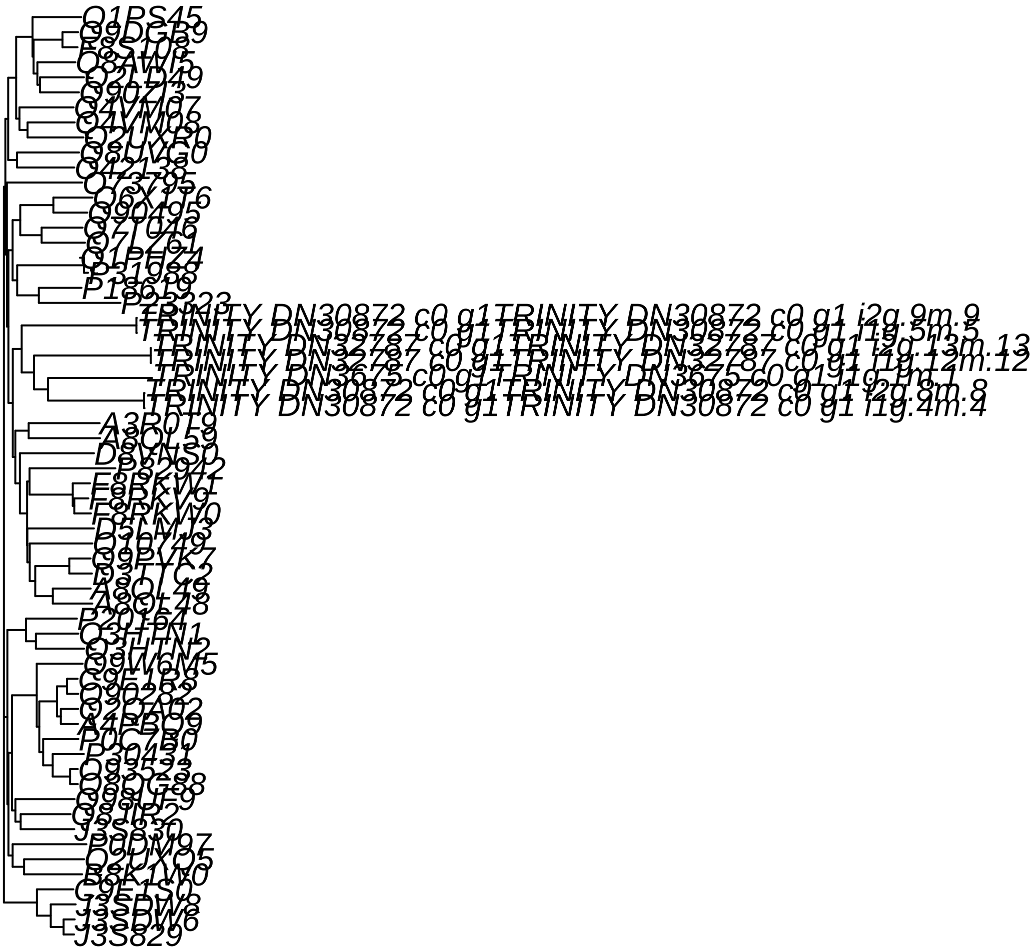

Q1PS45
Q9DGB9
Q21D49
Q4VM07
Q4VM08
Q2UXR0
Q8UVG0
Q42138
O73795
Q6X116
Q90495
Q7LQ46
Q7LZ61
Q1PHZ4
P18619
P22323
TRINITY DN30872 c0 g1TRINITY DN30872 c0 g1 i2g.9m.9
TRINITY DN30872 c0 g1TRINITY DN30872 c0 g1 i1g.5m.5
TRINITY DN32787 c0 g1TRINITY DN32787 c0 g1 i2g.13m.13
TRINITY DN32787 c0 g1TRINITY DN32787 c0 g1 i1g.12m.12
TRINITY DN3675 c0 g1TRINITY DN3675 c0 g1 i1g.1m.1
TRINITY DN30872 c0 g1TRINITY DN30872 c0 g1 i2g.8m.8
TRINITY DN30872 c0 g1TRINITY DN30872 c0 g1 i1g.4m.4
A3R0T9
A8QL59
D8VNS0
P82942
F8RKW1
F8RKV9
F8RKW0
D5LMJ3
Q9PVK7
B3TYC2
A8QL49
A8QL48
P20164
Q3HTN1
Q3HTN2
Q9W6M5
A4PBQ9
P0C7B0
Q8AUF9
Q8JIR2
J3S830
Q2UXQ5
B5K1W0
C9E1S0
J3SBW6
J3S829
